# Tendon gel using the film model method promotes ligament healing in rabbits

**DOI:** 10.1007/s00402-026-06280-w

**Published:** 2026-04-17

**Authors:** Rikuto Yoshimizu, Junsuke Nakase, Kazuaki Yoshioka, Toru Kuzumaki, Kojun Torigoe, Satoru Demura

**Affiliations:** 1 Kanazawa University, Kanazawa, Japan; 2 Department of Orthopaedics, Fukui General Hospital, Fukui, Japan; 3 Kanazawa University Graduate School of Medical Sciences, Kanazawa, Japan; 4 Tokai University, Hiratsuka, Japan; 5 Fukui Health Science University, Fukui, Japan

**Keywords:** Knee, Ligament biology and healing, Tendon biology and healing, Knee medial collateral ligament

## Abstract

**Introduction:**

Tendon gel is a translucent gel-like material secreted from the ends of a severed tendon. When mechanical stress is applied to a 3-day in vivo-preserved tendon gel, it matures into type I collagen–dominant tissue similar to normal tendon. This study aimed to evaluate the effects of transplanting a 3-day in vivo-preserved tendon gel into a knee medial collateral ligament (MCL) injury site in rabbits to promote intrinsic ligament regeneration.

**Materials and methods:**

Tendon gel was prepared from rabbit Achilles tendons using the film model method and harvested after 3 days of in vivo preservation. The 3-day tendon gel was transplanted into the knee MCL injury sites in another set of rabbits (*n* = 48). Additionally, the healing process was assessed at 1, 2, and 4 weeks postoperatively using mechanical and histological analyses. Ultimate load, peak stress, and elastic modulus were measured. Histological maturity was semi-quantitatively scored, and collagen type I and III expressions were examined by immunofluorescence staining.

**Results:**

At 2 weeks, the tendon gel group demonstrated significantly higher ultimate load than the control group (12.25 ± 4.90 vs. 5.25 ± 2.40 N; *p* = 0.02). The tendon gel group had greater peak stress than the control group (3.54 ± 1.44 vs. 1.69 ± 0.78 MPa; *p* = 0.02). Histological scores were higher in the tendon gel group than in the control group (7.25 ± 0.43 vs. 5.50 ± 1.73; *p* = 0.03). Cells in the tendon gel group were aligned parallel to collagen fibers with elongated nuclei, while type I collagen expression was stronger than that observed in controls.

**Conclusions:**

Transplanting a 3-day in vivo-preserved tendon gel into an injured ligament enhanced mechanical strength and histological maturation at 2 weeks postoperatively. These findings suggest that this tendon gel serves as a promising biomaterial for accelerating ligament healing.

## Introduction

The healing process of ligament injuries is categorized into the inflammatory, proliferative, and remodeling phases. Inflammatory cell infiltration and neovascular development occur during the inflammatory and proliferative phases, forming granulation tissue via mobilized fibroblasts [1, 2]. Collagen fibers in the healing region, arranged in the direction of stress, gain strength during remodeling via the replacement of type III collagen with type I collagen [2]. Injured ligament regeneration involves intrinsic and extrinsic regenerative processes [3]. The extrinsic regenerative process characterized by inflammatory cell infiltration and neovascular development during the inflammatory and proliferative phases has made regenerative medicine, which aims to adjust these processes to promote healing, a significant area of research attention. Tendons and ligaments share a highly aligned collagenous extracellular matrix and a common healing program: fibroblast-driven remodeling with early type III collagen progressively replaced by type I collagen [2]. Despite differences in vascularity and loading, collagen fibers align along the dominant tensile direction and gain strength as cross-links increase [2]. Accordingly, a tendon-derived material that captures intrinsic regenerative activity may provide a biologically relevant microenvironment to accelerate early ligament healing. Mesenchymal stem/stromal cell-based therapy has been explored as a regenerative approach that may augment conservative treatment for ligament injuries [4–8]. However, most strategies to enhance ligament healing primarily target extrinsic processes, including biologic augmentation with cells or growth factors, and mechanical support using synthetic or bioabsorbable scaffolds [9]. Although these approaches can facilitate defect bridging, they do not specifically isolate or harness the intrinsic regenerative program of dense collagenous tissues. Accordingly, a key gap remains in developing an intrinsic regenerative activity-derived biomaterial that can drive early maturation toward a type I collagen–dominant, tendon/ligament-like matrix [9].

Torigoe et al. developed the film model method, which enables the extraction of only the intrinsic regenerative process of tendons by placing the end of a severed mouse Achilles tendon between two thin films [10, 11]. Tendon gel, which is a translucent gel-like substance, is secreted from severed tendon sections. This gel matures physiologically and histologically when mechanical stress is applied, and its properties vary depending on the in vivo preservation period [12]. Shimozaki et al. investigated the in vivo preservation period of tendon gels. Type I collagen-like normal tendon tissue regenerates when mechanical stress is applied to a 3-day in vivo-preserved tendon gel, indicating the gel’s potential as a new biomaterial for treating soft-tissue injuries [13].

Ligament injuries are treated based on their severity and the presence of other concomitant injuries, with conservative treatment indicated for most minor or moderate injuries [14]. Joint mobilization training should be initiated as early as possible during rehabilitation since it delays ligament healing [14]. However, healed ligaments require a long time to recover their original properties following conservative treatment. Medial collateral ligament (MCL) injuries of the knee require more than 1 year to regain their original mechanical and histological properties after injury [15]. Elite athletes with MCL injuries return to sports within approximately 30 days, with few experiencing re-injury or significant performance loss [16]. Therefore, the most important therapeutic aspect is how safely and early return to sports can be accomplished in patients with minor or moderate ligament injuries that heal with conservative treatment.

The intrinsic regeneration of damaged tissue relies solely on its inherent potential. In this study, a 3-day in vivo-preserved tendon gel was transplanted into an injured knee MCL to enhance intrinsic regeneration, with the aim of evaluating its effects on ligament healing. We hypothesized that a 3-day in vivo-preserved tendon gel would promote the healing of an injured knee MCL.

## Materials and methods

All animal experiments were approved by the Institutional Animal Care and Use Committee and were conducted in accordance with institutional animal experiment regulations (approval number AP-194093).

Seventy-two skeletally mature Japanese White rabbits (15–17 weeks old; Kitayama Lab, Nagano, Japan) with a mean body weight of 3.2 (range, 3.0–3.5) kg were used in this study. They were housed individually in cages under controlled environmental conditions (temperature, 23 ± 3 °C; humidity, 55 ± 10%; 12-h light/dark cycle). Food and water were provided ad libitum, and postoperative activity, feeding, and wound condition were monitored daily. Of these, 24 rabbits were used exclusively for harvesting tendon gel from both Achilles tendons, yielding 48 tendon gel samples. The remaining 48 rabbits were used to create the knee MCL injury model. In these animals, one knee received tendon-gel transplantation (tendon gel group), whereas the contralateral knee was closed without tendon gel (control group). Each rabbit provided paired samples for within-animal comparison. Tendon gel harvesting and transplantation were performed on the same day to ensure consistent material quality and biological viability.

### Film model method and collection of the 3-day in vivo-preserved tendon gel

**Fig. 1:**
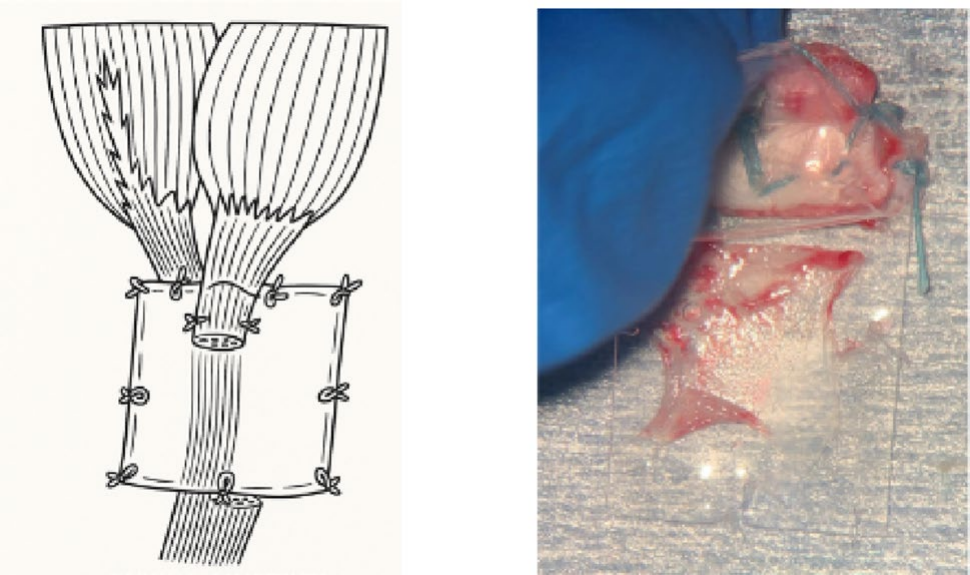
Procedure for creating tendon gel. A schematic diagram of the film model method extracted from the medial head of the gastrocnemius tendon (left) and the tendon gel after 3 days of in vivo preservation (right). This figure was newly created by the authors based on the concept of the film model previously described by Shimozaki et al. [13] and is presented solely for illustrative purposes

The Achilles tendon was severed near the insertion of the medial head of the gastrocnemius muscle into the calcaneus through a posteromedial skin incision. Rabbits used for tendon gel harvesting were distinct from those used to create the knee MCL injury model. The severed tendon proximal end was placed between two sheets of thin fluorine film (25 μm, 15 × 20 mm; Aflex 25 N NT, Asahi Glass, Tokyo, Japan), followed by fixation of the film-tendon complex with non-absorbable sutures. An additional film layer was applied over the sutures, and the film edges were secured with at least eight stitches. This configuration blocked extrinsic regenerative factors, isolating intrinsic regenerative activity as the tendon was completely sandwiched between impermeable fluorinated films. Subsequently, the film construct was reimplanted in vivo, and the wound was closed. Tendon gel was harvested after 3 days of in vivo preservation (Fig. 1), following the method described by Shimozaki et al. [13].

### Tendon gel transplantation into the knee medial collateral ligament injury model

**Fig. 2:**
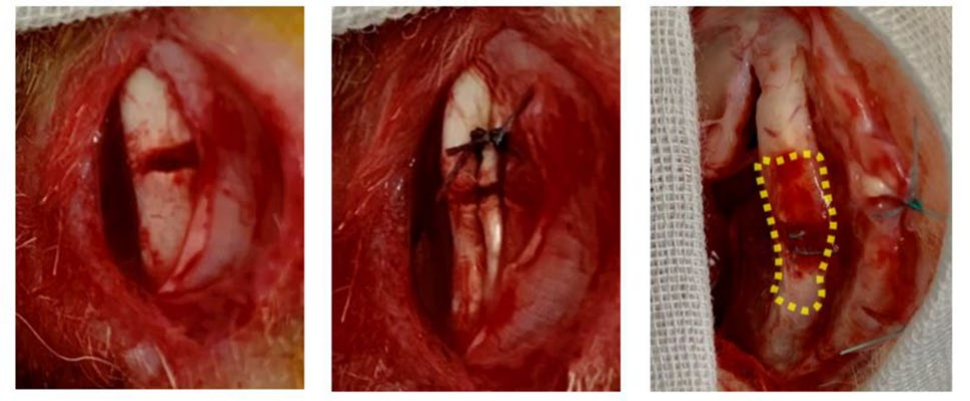
MCL injury model and tendon gel transplantation into the injury model. The knee MCL injury model was created by cutting the posterior two-thirds of the central knee MCL and suturing it with two non-absorbable surgical sutures (left, center). In the tendon gel group, a 3-day in vivo-preserved tendon gel extracted from another rabbit was implanted between the sutures. The knee MCL samples were harvested within the dotted area at 1, 2, and 4 weeks postoperatively (right). MCL, medial collateral ligament

The knee MCL injury model was established in rabbits, distinct from those used for tendon gel harvesting. A longitudinal incision was made on the medial aspect of the knee, and the MCL was exposed via blunt dissection. The posterior two-thirds of the central knee MCL were transected and repaired with two non-absorbable sutures (Fig. 2). A 3-day in vivo-preserved tendon gel was implanted between the sutures in one knee (tendon-gel group), whereas the contralateral knee was closed without tendon gel (control group). The assignment of tendon gel and control sides (right or left knees) was randomized preoperatively using a computer-generated random number sequence. All mechanical and histological evaluations were performed by independent investigators who were blinded to group allocation. Tendon gel harvesting and transplantation were conducted on the same day. Sixteen knee MCL specimens per group were harvested from each model at 1, 2, and 4 weeks postoperatively. Among these, eight were used for mechanical testing, and the remaining eight were used for histological analysis (Table 1).

**Table 1:** Animal allocation, sample collection, and downstream processing

Experiment / purpose		Rabbits (*n*)		Tissue / site	Key procedure			Downstream processing / endpoints		
Tendon gel preparation (film model)		24		Achilles tendons (bilateral)	Achilles tendon transection → proximal stump sandwiched between fluorine films → in vivo preservation for 3 days → gel harvested			Tendon gel samples used for transplantation (same day as harvest)		
Rabbit knee MCL injury model		48		Knee MCL (bilateral)	Mid-substance MCL partial transection (posterior two-thirds) + suture repair in both knees; 3-day tendon gel was implanted in one knee, whereas the contralateral knee received suture repair alone and was closed without tendon gel (side randomized).			Harvest at 1, 2, and 4 weeks; mechanical and histological analyses		

Time point	Rabbits (n)		Tendon gel knees (n)			Control knees (n)	Mechanical analysis (n/group)		Histological analysis (n/group)	
1 week	16		16			16	8		8	
2 weeks	16		16			16	8		8	
4 weeks	16		16			16	8		8	
Total	48		48			48	24		24	

Mechanical specimens were snap-frozen and tested via uniaxial tensile testing; histology specimens were formalin-fixed and processed for HE staining and collagen I/III immunofluorescence. HE, hematoxylin and eosin; MCL, medial collateral ligament

### Mechanical analysis

Samples for mechanical analysis were frozen in liquid nitrogen immediately after harvest and stored at − 80 °C. Before testing, the samples were thawed in phosphate-buffered saline at room temperature for 3 h. Next, the anterior one-third of each specimen was trimmed to isolate the healed region. The remaining tissue was shaped to dimensions of 15 mm in length, 3 mm in width, and 1 mm in thickness. Each specimen was mounted using a custom-made clamp, with an 8-mm segment of the healed region centered between the grips.

**Fig. 3:**
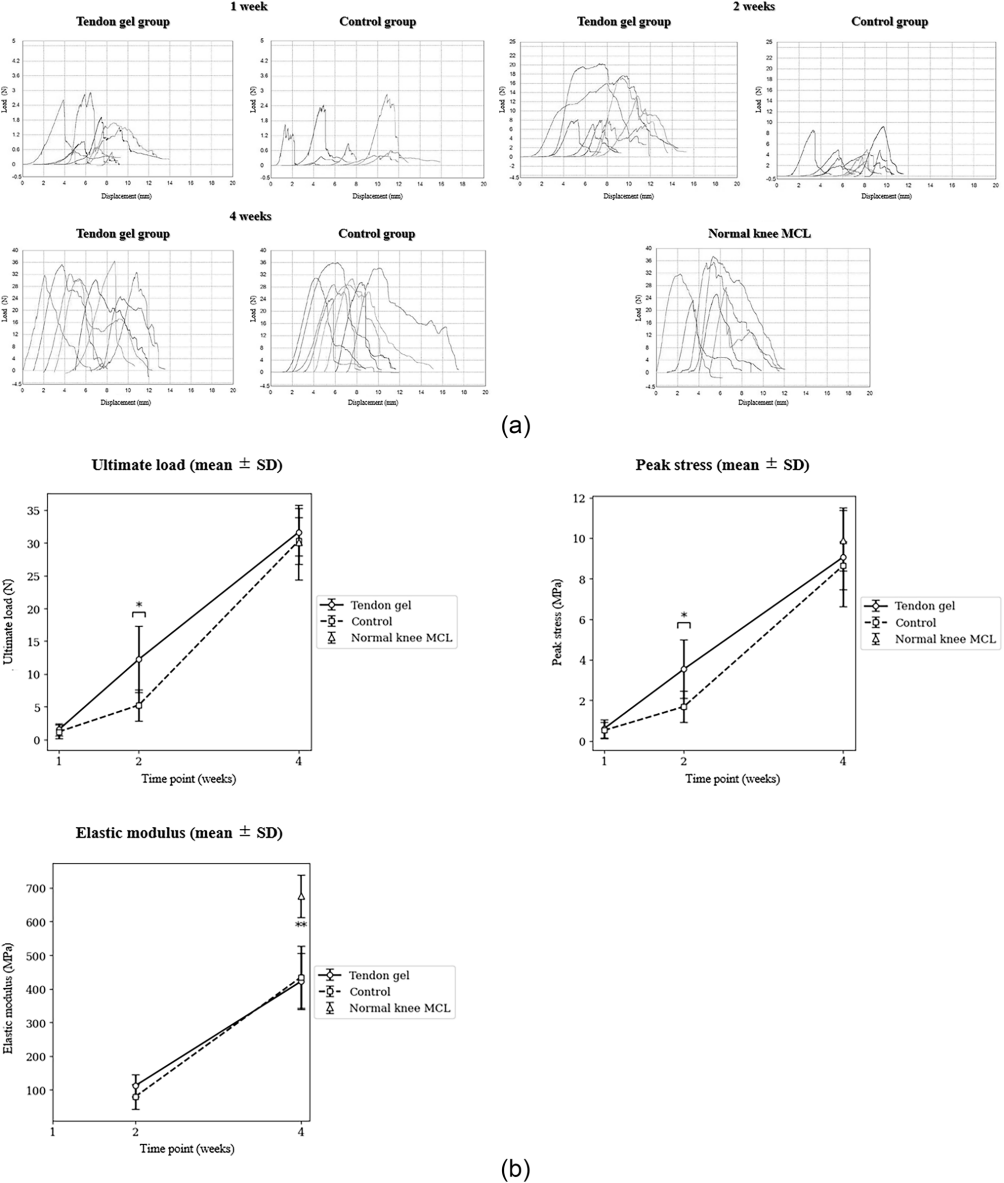
Mechanical analysis results. The vertical and horizontal axes indicate load (N) and displacement (mm), respectively. The upper row shows data from 1 and 2 weeks postoperatively, while the lower row shows data from 4 weeks postoperatively and normal knee MCL data. MCL, medial collateral ligament

Mechanical testing was performed using a universal testing machine (Autograph AG-X Plus 1 kN; Shimadzu Corp., Kyoto, Japan). Each specimen was preconditioned with five loading–unloading cycles between 0 and 0.5 N at a rate of 2.0 mm/min to minimize viscoelastic effects. After preconditioning, the specimen was held at 0.5 N for 10 s and subsequently loaded to failure at a constant crosshead speed of 2.0 mm/min (Fig. 3). Load–displacement data were recorded and converted to stress–strain curves using the initial cross-sectional area.

The ultimate load was defined as the maximum load before failure, while the peak stress was calculated by dividing the ultimate load by the initial cross-sectional area. Additionally, the elastic modulus was determined as the slope of the linear portion of the stress–strain curve, representing the tissue stiffness. All mechanical parameters were analyzed using TRAPEZIUMX-V software (Shimadzu Corp., Kyoto, Japan).

### Histological analysis

Harvested specimens were fixed in 10% neutral-buffered formalin, dehydrated through graded ethanol, and embedded in paraffin. Longitudinal sections were stained with hematoxylin and eosin to evaluate cellular morphology and collagen fiber organization.

Histological evaluation was performed using a semiquantitative scoring system modified from Xu et al. [17] for ligament tissue. Nine histological parameters were assessed to characterize tissue organization and cellular morphology. These included organization of the extracellular matrix, cell density and cell–matrix ratio, cell alignment, cell distribution within the repair tissue, cell nucleus morphology, organization of the repair tissue within the ligament callus, continuity between repaired and normal regions, vascularization within the defect, and degree of inflammation. Each parameter was graded from 0 to 2, except for vascularization and inflammation, which were graded from 0 to 1. The total histological score was calculated as the sum of all items, with a maximum possible score of 14 points representing the most mature healing.

The samples were stained using a standard immunostaining protocol as previously described [18]. Sections were deparaffinized, and rehydrated through graded alcohols, followed by antigen retrieval in DAKO Target Retrieval Solution (pH 9; Agilent Technologies, Santa Clara, CA, USA) at 95 °C for 30 min. Endogenous peroxidase activity was quenched after cooling, and non-specific binding was blocked using DAKO Protein Block (serum-free; Agilent). Sections were incubated overnight at 4 °C with the following primary antibodies: goat anti-collagen I (goat IgG; Southern Biotechnology, Birmingham, AL, USA) and mouse monoclonal anti-collagen III (clone FH-7 A, mouse IgG1; Abcam, Cambridge, UK). After rinsing, Alexa Fluor 488-conjugated anti-goat and Alexa Fluor 594-conjugated anti-mouse IgG (Thermo Fisher Scientific, Waltham, MA, USA) were applied as secondary antibodies for 1 h at room temperature. Nuclei were counterstained with 4′,6-diamidino-2-phenylindole (DAPI).

Confocal fluorescence images were obtained using an inverted confocal microscope (Eclipse Ti2; Nikon Instruments, Tokyo, Japan) equipped with an Andor Dragonfly spinning-disk unit and an EMCCD camera (iXon DU888; Andor Technology Ltd., Oxford Instruments, UK). Excitation wavelengths of 405 (DAPI), 488 (collagen I), and 594 (collagen III) were used. Type I collagen, type III collagen, and nuclei appeared green, red, and blue, respectively. All images were acquired under identical exposure and gain settings. Finally, collagen I/III distributions were qualitatively compared between groups.

### Statistical analysis

All statistical analyses were performed using IBM SPSS Statistics for Windows, version 23.0 (IBM Corp., Armonk, NY, USA). Data normality was assessed using the Shapiro–Wilk test. Since the tendon gel and control groups were obtained from contralateral limbs of the same rabbits, within-animal comparisons were analyzed using paired *t*-tests or Wilcoxon signed-rank tests for normally and non-normally distributed data, respectively. Comparisons were conducted separately for each postoperative time point (1, 2, and 4 weeks). Holm-adjusted *p*-values were calculated to control for type I error across multiple comparisons at each time point. Comparisons with normal knee MCL specimens were performed using Welch’s t-test (unequal variances), since normal samples were independent of the paired injury specimens.

Effect sizes were expressed as Cohen’s *dₓ*. Intra- and inter-observer reliabilities of histological scores were calculated using intraclass correlation coefficients (ICC) and categorized as poor (0.00–0.40), fair-to-good (0.41–0.75), or good-to-excellent (0.76–1.00). Intra-observer reliability was based on three repeated measurements obtained at 2-week intervals by observer 1 (RY), whereas inter-observer reliability was calculated from the first measurement of observer 1 and a single measurement of observer 2 (JN).

A priori power analysis based on previous tensile-testing data for rabbit knee MCLs (effect size = 1.54, α = 0.05, power = 0.80) indicated that eight paired specimens per group at each time point were required, which was achieved in this study [14].

## Results

### Mechanical analysis

At 2 weeks, the tendon gel knees demonstrated significantly higher ultimate load than the contralateral control knees (mean ± standard deviation, 12.25 ± 4.90 vs. 5.25 ± 2.40 N; *p* = 0.02). Peak stress similarly favored the tendon gel knees compared with the contralateral control knees (3.54 ± 1.44 vs. 1.69 ± 0.78 MPa; *p* = 0.02) (Table 2; Fig. 3). No between-group differences were observed at 1 or 4 weeks for load or stress. Elastic modulus showed no between-group difference at any time point (Table 2; Fig. 3). At 4 weeks, the elastic modulus of both groups remained significantly lower than that of normal knee MCL (tendon gel group: 422.00 ± 83.76 MPa; control group: 434.50 ± 91.99 MPa; normal: 675.87 ± 62.88 MPa; *p* = 3.0 × 10^− 5^ and 8.3 × 10^− 5^, respectively). However, ultimate load and peak stress in both groups did not significantly differ from those in the normal group (Table 2; Fig. 3).

**Table 2:** Mechanistic analysis results

	1 week				2 weeks			4 weeks			
	Tendon gel group	Control group	*p* value		Tendon gel group	Control group	*p* value	Tendon gel group		Control group	*p* value
Evaluation range	0.5–1.0 N				1.0–2.0 N			5.0–10.0 N			
Ultimate load (N)	1.51 ± 0.92	1.19 ± 1.01	0.51		**12.25 ± 4.90**	**5.25 ± 2.38**	**0.02**	31.65 ± 3.58		30.34 ± 3.57	0.81
Peak stress (MPa)	0.61 ± 0.45	0.52 ± 0.40	0.62		**3.54 ± 1.44**	**1.69 ± 0.78**	**0.02**	9.07 ± 2.44		8.65 ± 1.20	0.63
Elastic modulus (MPa)	—	—	—		112.33 ± 33.49	79.56 ± 36.95	0.13	422.00 ± 83.76		434.50 ± 91.99	0.73

Elastic modulus was not calculated at 1 week because a clearly defined linear region could not be identified. Evaluation range indicates the load range used to define the linear region for modulus calculation. Values are mean ± standard deviation. *p*-values are Holm-adjusted (paired comparisons). Statistical significance is defined as *p* < 0.05

### Histological analysis

**Fig. 4:**
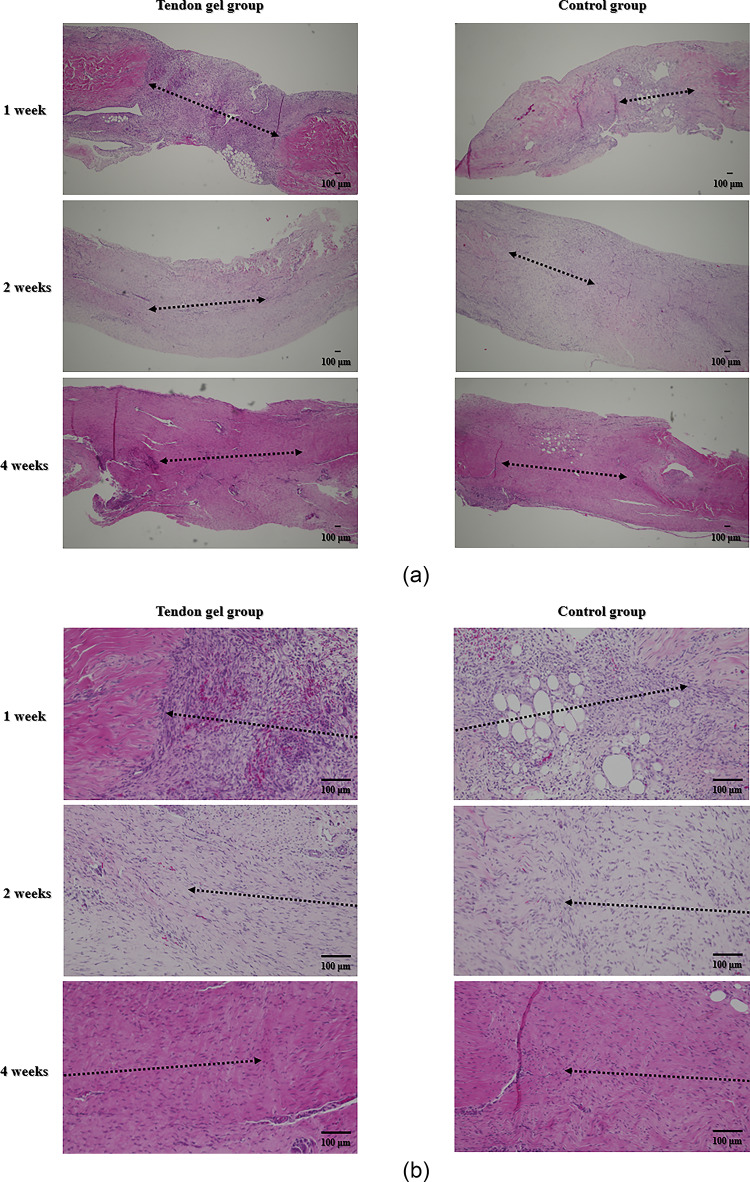
Evaluation of hematoxylin and eosin staining. (a) Low-magnification overview images of the tendon gel (left) and control (right) groups at 1, 2, and 4 weeks postoperatively. (b) Higher-magnification images of the healing region at the corresponding time points were used to visualize fibroblast morphology and extracellular matrix (ECM) organization/matrix formation. All specimens are arranged with the proximal and distal sides to the left and right, respectively. The bar indicates the healing area. Scale bars: 100 μm

At 1 week postoperatively, both groups exhibited poorly organized extracellular matrix with round cells distributed throughout the repair tissue. The border between the normal ligament and the healing region was distinct (Fig. 4). The mean histological score did not differ significantly between the tendon gel and control groups (2.13 ± 1.27 vs. 2.25 ± 1.12; *p* = 0.85) (Table 3).

**Table 3:** Histological scoring results

	1 week				2 weeks			4 weeks			
	Tendon gel group	Control group	*p* value		Tendon gel group	Control group	*p* value	Tendon gel group		Control group	*p* value
ECM organization of the whole ligament	0.88 ± 0.33	0.88 ± 0.33			2	1.75 ± 0.43	0.17	2		2	
Cellularity/cell-matrix ratio	0.25 ± 0.43	0.13 ± 0.33	0.55		1	0.88 ± 0.33	0.35	1.25 ± 0.43		1.13 ± 0.33	0.13
Cell alignment	0	0			**0.88 ± 0.33**	**0.38 ± 0.48**	**0.04**	2		2	
Cell distribution	0	0			0	0		0.25 ± 0.43		0.5 ± 0.5	0.33
Cell nucleus morphology	0	0			**1**	**0.5 ± 0.5**	**0.03**	1.38 ± 0.48		1.75 ± 0.43	0.15
Organization of repair tissue of the ligament callus	0.13 ± 0.33	0	0.35		1	0.88 ± 0.33	0.35	1.5 ± 0.5		1.5 ± 0.5	
Transition from defect to normal tissue	0.63 ± 0.48	0.63 ± 0.48			1.25 ± 0.43	0.88 ± 0.33	0.09	1.75 ± 0.43		1.38 ± 0.48	0.15
Vascularization in the defect area	0.25 ± 0.38	0.63 ± 0.48	0.15		0	0.25 ± 0.43	0.17	1		1	
Inflammation	0	0			0.13 ± 0.33	0	0.35	1		1	
Total score	2.13 ± 1.27	2.25 ± 1.12	0.85		7.25 ± 0.43	5.50 ± 1.73	**0.03**	12.13 ± 1.17		12.25 ± 1.56	0.87

Values are expressed as mean ± standard deviation. Scoring was based on a modified version of the system proposed by Xu et al. [17], adapted for ligament tissue. *p*-values were calculated using the Wilcoxon signed-rank test for paired comparisons and Holm-adjusted for multiple comparisons (Holm method). Statistical significance was set at *p* < 0.05. Each item was scored as indicated (0–2 or 0–1); the maximum total score was 14. ECM, Extracellular matrix

At 2 weeks, the tendon gel group showed more advanced tissue organization than the control group. Cells were aligned parallel to the collagen fibers, and their nuclei exhibited an elongated morphology, indicating early maturation of fibroblast-like cells. However, the control group retained round or irregular nuclei with less organized cellular alignment (Fig. 4). The mean histological score was significantly higher in the tendon gel group than in the control group (7.25 ± 0.43 vs. 5.50 ± 1.73; *p* = 0.03), indicating enhanced healing progression (Table 3).

At 4 weeks, the extracellular matrix was well organized in both groups, with spindle-shaped fibroblast-like cells arranged in a fibrous pattern and small blood vessels in the healing area (Fig. 4). No significant difference in total histological scores were found between the tendon gel and control groups (12.13 ± 1.17 vs. 12.25 ± 1.56; *p* = 0.87) (Table 3).

**Fig. 5:**
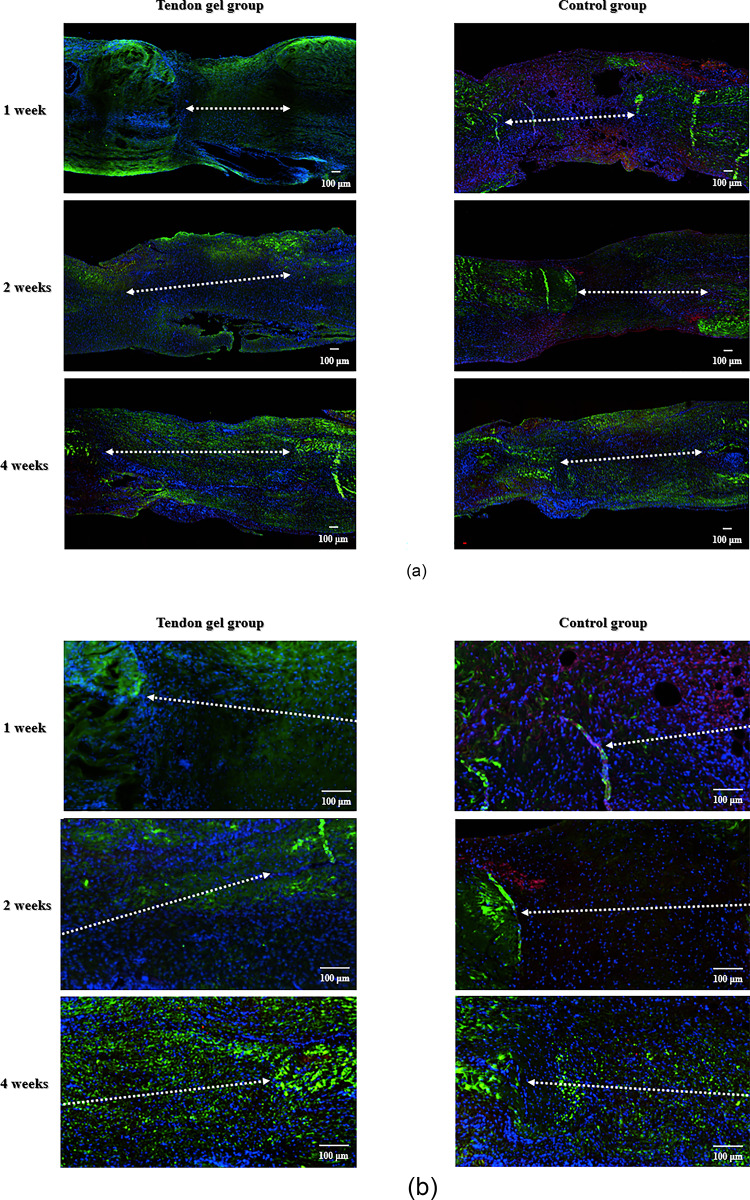
Immunohistological evaluation of types I and III collagen. (a) Low-magnification images of the tendon gel (left) and control (right) groups at 1, 2, and 4 weeks postoperatively. (b) Higher-magnification images of the healing region at the corresponding time points were used to better visualize collagen distribution and cellularity. Type I collagen, type III collagen, and cell nuclei are shown in green, red, and blue, respectively. All specimens are arranged with the proximal and distal sides to the left and right, respectively. The bar indicates the healing area. Scale bars: 100 μm

Immunostaining showed that type I collagen was strongly expressed at all time points in the tendon gel group compared with the control group, mainly in the peripheral region. Type III collagen was expressed in the first week in the control group, whereas type I collagen expression was not strong until 4 weeks postoperatively (Fig. 5).

The inter-observer (ICC: 0.90; 95% confidence interval [CI]: 0.86–0.93) and intra-observer (ICC: 0.81; 95% CI: 0.71–0.88) reliabilities of the histological scores were excellent at 2 weeks postoperatively.

## Discussion

In this study, a 3-day in vivo-preserved tendon gel transplantation into a rabbit knee MCL injury site accelerated early ligament healing. Specifically, the tendon gel group demonstrated significantly higher ultimate load and peak stress and more advanced histological maturation than the control group at 2 weeks postoperatively, indicating that tendon gel promotes intrinsic regeneration of ligament tissue. No between-group differences were detected at 1 or 4 weeks. At 4 weeks, ultimate load and peak stress in both groups approached those of normal knee MCL, whereas the elastic modulus remained substantially lower than normal, suggesting incomplete recovery of tissue stiffness despite failure strength restoration. These findings align with previous ones demonstrating successful ligament regeneration using bioabsorbable scaffolds in rabbit models [19].

To interpret these findings, Shimozaki et al. used a rabbit model to investigate histological and structural differences before and after tensile testing of tendon gel generated under different preservation periods (3, 5, 10, and 15 days) [13]. They reported that 3-day in vivo-preserved tendon gels regenerated thick type I collagen fibers comparable to those of normal tendons, with more molecular cross-links than other tendon gels after tensile stress. Based on these findings, we hypothesized that a 3-day in vivo-preserved tendon gel could facilitate soft-tissue regeneration, particularly ligament healing, through its intrinsic regenerative potential.

In this study, the ultimate load of the tendon gel group was approximately 2.3 times higher than that of the control group at 2 weeks postoperatively. Fibroblasts migrate to the injured area in both ligaments and tendons during healing. Newly synthesized procollagen is processed and assembled into collagen fibrils, and covalent cross-linking promotes fibril stabilization and fiber formation [20–22]. Shimozaki et al. found that 3-day in vivo-preserved tendon gels had characteristic circular fibroblasts and contained abundant tropocollagen-related components before tensile loading [13]. This feature of the tendon gel may have encouraged the intermolecular cross-linking of collagen in the healing area, influencing the mechanical analysis results. Normal knee MCL specimens were also tested and compared with the 4-week repairs. The ultimate load, peak stress, and elastic modulus of the normal knee MCL were 30.07 ± 5.71 N, 9.90 ± 1.49 MPa, and 675.87 ± 62.88 MPa, respectively (Fig. 3). At 4 weeks, the ultimate load in both groups was comparable to that of normal knee MCL; however, the elastic modulus was approximately 65% (tendon gel group: *p* < 0.01, control group: *p* = 0.01). This discrepancy suggests that failure strength recovery precedes tissue stiffness recovery during ligament remodeling. Ultimate load reflects the maximum force the tissue can withstand at failure, whereas elastic modulus is strongly influenced by the collagen network quality, including fiber continuity, crimp morphology, alignment, and cross-link density [20, 22–25]. Therefore, incomplete maturation of the collagen architecture may continue to limit stiffness even if sufficient collagen mass and gross bridging restore failure strength by 4 weeks. This interpretation aligns with previous observations demonstrating that ligament stiffness and microstructural organization can remain impaired long after apparent recovery of failure load [15].

During healing, collagen production and reorganization occur from the proliferation phase to the remodeling phase, resulting in a progressive decrease in cellularity and an increase in fibrous matrix. Collagen fibers align with the tensile stress direction. This process is accompanied by a decrease in type III collagen, vascularization, and cellular components and a progressive replacement with type I collagen with increased cross-linking and tensile strength [1, 2]. The cell nucleus shape in the healing area flattens as it matures, resembling the elongated nuclei observed in native tendon/ligament tissue [12]. In our histological analysis, cells with elongated, heterochromatic nuclei were aligned parallel to the collagen fibers at 2 weeks in the tendon gel group, indicating more advanced histological maturation. Type I collagen expression also appeared stronger in the tendon gel group than in the control group across time points. The organization of collagen fibers and restoration of type I collagen concentration are crucial factors during the healing of injured ligaments and tendons, as defects in these factors reduce the healed area’s mechanical strength [1, 2].

In summary, early physiological loading during postoperative activity may have promoted more structured alignment and cross-linking of type I collagen molecules in the healing area during the first 2 weeks postoperatively in the tendon gel group, thereby improving mechanical strength. The time-dependent pattern observed in this study aligns with the phase-specific biology of ligament healing [1, 2]. At 1 week, the repair tissue is dominated by inflammatory and early proliferative responses, with high cellularity and limited matrix organization, possibly masking group-dependent differences in mechanical performance and histological maturity [1]. By 2 weeks, the tissue transitions toward early remodeling, during which collagen alignment and maturation become key determinants of strength [1, 2]. The tendon gel may be particularly effective during this window by providing an intrinsically derived matrix environment that promotes earlier organization toward a type I collagen-dominant, aligned structure. Ongoing endogenous remodeling in both groups may partially “catch up” by 4 weeks, reducing between-group differences in gross strength and histological scores [1, 2].

Tendon gel is a substance that is secreted from the cut tendon stump. Tendons and ligaments share key structural and biological features and undergo broadly similar healing phases, supporting the rationale for transplanting tendon gel to injured ligaments [2, 23]. Ligament reconstruction using tendons is the most common procedure for ligament injuries, including those of the anterior cruciate ligament. In this study, a gel-like substance was observed at transected knee MCL ends, although it was less abundant than that derived from the Achilles tendon and may provide less resistance to early mechanical loading. This difference may reflect the tendon’s high regenerative capacity; however, the size of the cut stump may also influence the amount of secreted gel. The limited amount of ligament-derived gel underscores the potential value of tendon gel as a biomaterial.

A key feature of this study is the use of gel-like secretory tissue derived from intrinsically regenerating tendon as a biomaterial to enhance ligament healing. These findings support further development of tendon gel–based biomaterials for soft-tissue repair. However, immunological safety and standardization will be important considerations for clinical translation, and an engineered tendon gel mimic may be preferable. An injectable formulation of such a mimic may be particularly advantageous for clinical application. Compositional analysis of the 3-day in vivo-preserved tendon gel demonstrated that it was composed of tendon-derived cells and collagens I, III, and V, with approximate proportions of 41%, 33%, and 26%, respectively. Future studies should identify bioactive mediators (e.g., growth factors) within tendon gel and determine which components are necessary and sufficient to reproduce its effects.

This study has some limitations. First, a mid-substance (parenchymal) knee MCL injury model was used, whereas many clinical knee MCL injuries involve the ligament–bone interface (enthesis) [23–26]. Insertional healing occurs in a distinct biological and mechanical environment, including fibrocartilaginous transition zones, mineralized tissue, and different stress distributions, possibly leading to healing responses that differ from mid-substance repair [23–26]. Although this parenchymal model enabled observation of intrinsic connective-tissue healing, translational relevance to insertional healing should be cautiously interpreted. Nevertheless, the parenchymal design provided a reproducible and controllable environment to assess the gel’s intrinsic effects on early matrix organization and maturation. Partial lacerations were sutured to prevent complete rupture during harvesting, as unsutured partial tear models frequently failed before analysis. Second, only uniaxial tensile testing was performed, even though physiological loading on the ligament is multidirectional. Three-dimensional loading models or finite-element analyses may provide additional insight into the healing ligament’s biomechanical behavior. However, uniaxial testing was selected to reduce variability arising from differences in specimen geometry and insertion morphology, allowing a focused assessment of intrinsic material properties [24]. Third, histological evaluation was limited to type I and III collagen expression and semiquantitative scoring. Other extracellular matrix components, such as proteoglycans (e.g., decorin and fibromodulin) and type V collagen, which regulate collagen fibrillogenesis and maturation, were not analyzed. Future studies using quantitative immunohistochemistry and molecular assays are needed to comprehensively elucidate the compositional remodeling process [25]. Fourth, this study used rabbits, which exhibit prolonged knee flexion (> 90°) and faster metabolic turnover than humans. These anatomical and physiological differences may influence tissue healing dynamics; therefore, extrapolation to the human clinical setting should be made cautiously. Finally, the potential immune response to the transplanted material was not investigated. Although the tendon gel was allogeneic, future translational applications should evaluate the biocompatibility and immunological safety of any synthetic or allogeneic mimics. Despite these limitations, this study’s findings demonstrate that a 3-day in vivo-preserved tendon gel enhanced ligament healing and may serve as a foundation for developing biomaterial-based regenerative therapies.

In conclusion, application of tendon gel to the injured knee MCL resulted in significantly higher mechanical strength (ultimate load and peak stress) and histological scores at 2 weeks postoperatively than no treatment, indicating that tendon gel promotes healing of the injured area. These findings provide new insights into potential treatment options for ligament injuries.

## Data Availability

The datasets generated and analyzed during the current study are available from the corresponding author on reasonable request.

## References

[1] Chamberlain CS, Crowley E, Vanderby R (2009) The spatio-temporal dynamics of ligament healing. Wound Repair Regen 17:206–215. 10.1111/j.1524-475X.2009.00465.x. PubMed PMID: 19320889 19320889 10.1111/j.1524-475X.2009.00465.x PMC3214965

[2] Leong NL, Kator JL, Clemens TL, James A, Enamoto-Iwamoto M, Jiang J (2020) Tendon and ligament healing and current approaches to tendon and ligament regeneration. J Orthop Res 38:7–12. 10.1002/jor.24475PubMed PMID: 31529731 31529731 10.1002/jor.24475 PMC7307866

[3] Titan AL, Foster DS, Chang J, Longaker MT (2019) Flexor tendon: development, healing, adhesion formation, and contributing growth factors. Plast Reconstr Surg 144:639e–647. 10.1097/PRS.0000000000006048PubMed PMID: 31568303 e 31568303 10.1097/PRS.0000000000006048 PMC7092377

[4] Han X, Liao R, Li X, Zhang C, Huo S, Qin L et al (2025) Mesenchymal stem cells in treating human diseases: molecular mechanisms and clinical studies. Signal Transduct Target Ther 10:262. 10.1038/s41392-025-02313-9PubMed PMID: 40841367 40841367 10.1038/s41392-025-02313-9 PMC12371117

[5] Meirelles LD, Fontes AM, Covas DT, Caplan AI (2009) Mechanisms involved in the therapeutic properties of mesenchymal stem cells. Cytokine Growth Factor Rev 20:419–427. 10.1016/j.cytogfr.2009.10.002PubMed PMID: 19926330 19926330 10.1016/j.cytogfr.2009.10.002

[6] Saether EE, Chamberlain CS, Leiferman EM, Kondratko-Mittnacht JR, Li WJ, Brickson SL, Vanderby R (2014) Enhanced medial collateral ligament healing using mesenchymal stem cells: dosage effects on cellular response and cytokine profile. Stem Cell Rev Rep 10:86–96. 10.1007/s12015-013-9479-7PubMed PMID: 24174129 24174129 10.1007/s12015-013-9479-7 PMC3946728

[7] Tei T, Matsumoto K, Mifune Y, Ishida K, Sasaki K, Shoji T et al (2008) Administrations of peripheral blood CD34-positive cells contribute to medial collateral ligament healing via vasculogenesis. Stem Cells 26:819–830. 10.1634/stemcells.2007-0671. PubMed PMID: 18192236 18192236 10.1634/stemcells.2007-0671

[8] Jiang D, Yang S, Gao P, Zhang Y, Guo T, Lin H, Geng H (2015) Combined effect of ligament stem cells and umbilical-cord-blood-derived CD34 + cells on ligament healing. Cell Tissue Res 362:587–595. 10.1007/s00441-015-2250-4Epub. PubMed PMID: 26224540 26224540 10.1007/s00441-015-2250-4

[9] Tang Y, Wang Z, Xiang L, Zhao Z, Cui W (2022) Functional biomaterials for tendon/ligament repair and regeneration. Regen Biomater 9:rbac062. 10.1093/rb/rbac062. PubMed PMID: 36176715 36176715 10.1093/rb/rbac062 PMC9514853

[10] Torigoe K, Hashimoto K, Lundborg G (1999) A role of migratory Schwann cells in a conditioning effect of peripheral nerve regeneration. Exp Neurol 160:99–108. 10.1006/exnr.1999.7202. PubMed PMID: 10630194 10630194 10.1006/exnr.1999.7202

[11] Torigoe K, Tanaka HF, Yonenaga K, Ohkochi H, Miyasaka M, Sato R et al (2011) Mechanisms of collagen fibril alignment in tendon injury: from tendon regeneration to artificial tendon. J Orthop Res 29:1944–1950. 10.1002/jor.21460PubMed PMID: 21618275 21618275 10.1002/jor.21460

[12] Ohashi Y, Nakase J, Shimozaki K, Torigoe K, Tsuchiya H (2018) Evaluation of dynamic change in regenerated tendons in a mouse model. J Exp Orthop 5:37. 10.1186/s40634-018-0152-6PubMed PMID: 30242576 30242576 10.1186/s40634-018-0152-6 PMC6150864

[13] Shimozaki K, Nakase J, Ohashi Y, Kuzumaki T, Yamaguchi T, Torigoe K, Tsuchiya H (2022) Investigating the histological and structural properties of tendon gel as an artificial biomaterial using the film model method in rabbits. J Exp Orthop 9:1. 10.1186/s40634-021-00434-y, PubMed PMID: 34978637 10.1186/s40634-021-00434-y PMC8724385 34978637

[14] Thornton GM, Johnson JC, Maser RV, Marchuk LL, Shrive NG, Frank CB (2005) Strength of medial structures of the knee joint are decreased by isolated injury to the medial collateral ligament and subsequent joint immobilization. J Orthop Res 23:1191–1198. 10.1016/j.orthres.2005.03.002PubMed PMID: 16140200 16140200 10.1016/j.orthres.2005.03.002

[15] Woo SL, Inoue M, McGurk-Burleson E, Gomez MA (1987) Treatment of the medial collateral ligament injury. II: Structure and function of canine knees in response to differing treatment regimens. Am J Sports Med 15:22–29. 10.1177/036354658701500104PubMed PMID: 3812858 3812858 10.1177/036354658701500104

[16] Lavoie-Gagne OZ, Retzky J, Diaz CC, Mehta N, Korrapati A, Forlenza EM et al (2021) Return-to-play times and player performance after medial collateral ligament injury in elite-level European soccer players. Orthop J Sports Med 9:23259671211033904. 10.1177/23259671211033904PubMed PMID: 34604429 34604429 10.1177/23259671211033904 PMC8485161

[17] Xu W, Wang Y, Liu E, Sun Y, Luo Z, Xu Z et al (2013) Human iPSC-derived neural crest stem cells promote tendon repair in a rat patellar tendon window defect model. Tissue Eng Part A 19:2439–2451. 10.1089/ten.TEA.2012.0453. PubMed PMID: 23815150 23815150 10.1089/ten.tea.2012.0453 PMC3807699

[18] Yoshioka K, Yoshida K, Cui H, Wakayama T, Takuwa N, Okamoto Y et al (2012) Endothelial PI3K-C2α, a class II PI3K, has an essential role in angiogenesis and vascular barrier function. Nat Med 18:1560–1569. 10.1038/nm.2928. PubMed PMID: 22983395 22983395 10.1038/nm.2928

[19] Nishimoto H, Kokubu T, Inui A, Mifune Y, Nishida K, Fujioka H et al (2012) Ligament regeneration using an absorbable stent-shaped poly-l-lactic acid scaffold in a rabbit model. Int Orthop 36:2379–2386. 10.1007/s00264-012-1660-0. PubMed PMID: 22976595 22976595 10.1007/s00264-012-1660-0 PMC3479301

[20] Ellingson AJ, Pancheri NM, Schiele NR (2022) Regulators of collagen crosslinking in developing and adult tendons. Eur Cell Mater 43:130–152. 10.22203/eCM.v043a11. PubMed PMID: 35380167 35380167 10.22203/eCM.v043a11 PMC9583849

[21] Kuzumaki T, Yamazaki K, Suzuki K, Torigoe K (2017) Appropriate tensile mode and timing of applying tension to promote tendon gel regeneration. Tissue Eng Regen Med 14:465–475. 10.1007/s13770-017-0050-5PubMed PMID: 30603502 30603502 10.1007/s13770-017-0050-5 PMC6171615

[22] Kamml J, Acevedo C, Kammer DS (2023) Advanced-glycation endproducts: how cross-linking properties affect the collagen fibril behavior. J Mech Behav Biomed Mater 148:106198. 10.1016/j.jmbbm.2023.106198, PubMed PMID: 37608934 10.1016/j.jmbbm.2023.106198 PMC11519298 37890341

[23] Bobzin L, Roberts RR, Chen HJ, Crump JG, Merrill AE (2021) Development and maintenance of tendons and ligaments. Development 148:dev186916. 10.1242/dev.186916, PubMed PMID: 33913478 10.1242/dev.186916 PMC8077520 33913478

[24] Schmidt EC, Chin M, Aoyama JT, Ganley TJ, Shea KG, Hast MW (2019) Mechanical and microstructural properties of native pediatric posterior cruciate and collateral ligaments. Orthop J Sports Med 7:2325967118824400. 10.1177/2325967118824400PubMed PMID: 30775386 30775386 10.1177/2325967118824400 PMC6362518

[25] Chamberlain CS, Crowley EM, Kobayashi H, Eliceiri KW, Vanderby R (2011) Quantification of collagen organization and extracellular matrix factors within the healing ligament. Microsc Microanal 17:779–787. 10.1017/S1431927611011925. PubMed PMID: 21910939 21910939 10.1017/S1431927611011925 PMC3263369

[26] Xu Z, Xu W, Zhang T, Luo L (2024) Mechanisms of tendon-bone interface healing: biomechanics, cell mechanics, and tissue engineering approaches. J Orthop Surg Res 19:817. 10.1186/s13018-024-05304-8PubMed PMID: 39623392 39623392 10.1186/s13018-024-05304-8 PMC11613615

